# Experimental benchmarking remains crucial to the interpretation of sequence annotation data

**DOI:** 10.1128/msystems.00772-26

**Published:** 2026-09-29

**Authors:** Claire M. Palmer

**Affiliations:** 1 Department of Bacteriology, University of Wisconsin–Madison 205263, Madison, Wisconsin, USA; Istanbul Medipol Universitesi Uluslararasi Tip Fakultesi, Istanbul, Turkey

**Keywords:** hopanoid

## Abstract

Determining the distribution and origin of bacterial synthesis of metabolites of interest relies on computational prediction of protein function. In their recent study, Lawrence and colleagues employed a rigorous pipeline for benchmarking their annotation methods (E. C. Lawrence, H. Pan, N. Ollikainen, and B. J. Belin, mSystems 11:e00619-26, 2026, https://doi.org/10.1128/msystems.00619-26). This allowed them to distinguish between related yet functionally distinct proteins, thereby refining their understanding of bacterial hopanoid production. They find that the ability to synthesize these compounds likely arose in marine alphaproteobacteria and is related to osmotic stress tolerance. This study additionally provides new insights into the extracellular transport of hopanoids. The combination of large-scale computational analysis with experimentally based benchmarking that they describe provides a useful model for any researcher interested in understanding bacterial biosynthetic capability.

## COMMENTARY

The abundance of currently available genomic data allows researchers to quickly and easily explore the biosynthetic capacity of a wide range of bacterial strains. Culture-independent methods are particularly valuable for assessing secondary metabolite production capability, as production of these compounds is often sensitive to environmental conditions (1). By evaluating the distribution of biosynthetic genes across species, researchers can develop hypotheses about the evolutionary history and environmental context of these metabolisms, identify strains for future experiments, and identify related genes for characterization.

But the quality of these inferences depends on the quality of our annotation methods, especially when we aim to determine which specific metabolites an organism can produce. There are a variety of approaches for computationally assigning predicted functions to amino acid sequences derived from genomic data (2). Hidden Markov models (HMMs) (3) are commonly used to build profiles for a given protein function that can be used to look for new sequences that may also perform that function (4, 5). However, related proteins may share features, such as a similar substrate-binding cavity, yet produce different classes of compounds (6). Experimental validation is key to unpicking these nuances, requiring rigorous benchmarking of our computational methods.

One area where there has been particular interest in surveying biosynthetic capacity is the class of specialized lipids called hopanoids (7). These planar hydrocarbons have five rings, a range of potential side chain modifications, and are found in the membranes of some bacterial species (8). While bacterial production of hopanoids was first observed in 1971 (9, 10), interest in these compounds predates this discovery. When hopanoids are degraded during rock formation, they lose many of their modifications, leaving behind hydrocarbon skeletons called hopanes (8). Geologists first detected hopanes in 1965 (11), and since then, there has been continued interest in using these compounds as a window into life on ancient Earth (7).

Since the initial discovery of bacterial hopanoid production, researchers have identified various enzymes involved in hopanoid biosynthesis. In order to produce hopanoids at all, species must have the ability to generate the characteristic pentacyclic backbone through the activity of squalene-hopene cyclase (SHC) (12). Following this cyclization, other enzymes deposit side chain modifications (8). Many of these side chains are lost during rock formation, leading to particular interest in the enzymes that produce hopanoid modifications observed in the rock record (7). The enzyme HpnH adds adenosine to the hopanoid backbone, the first step in the production of extended C_35_ hopanoids (13). Hopanoids can also be methylated, with HpnP responsible for C2-methylation (14) and HpnR for C3-methylation (15). There is interest in understanding the distributions not only of hopanoids as a class but also of these different modified versions, with particular controversy surrounding the origin of 2-methylation capacity (16, 17).

In a recent study in *mSystems*, Lawrence et al. sought to understand the origins of hopanoid synthesis by analyzing a library of 62,143 microbial genomes (18). The authors began by benchmarking available HMM profiles using experimental data. They collected protein sequences for SHC, HpnP, HpnR, and HpnH that had been experimentally validated by genetic or *in vitro* studies. They then expanded this data set by incorporating homologous sequences from the genomes of all strains experimentally observed to produce hopanoids, or, in the case of modification enzymes, a specific type of hopanoid. In addition to this positive control set of sequences, they also assembled sequences from related proteins that produce and/or act on different chemicals for use as negative controls. Lawrence and colleagues found that the most reliable profiles for each enzyme came from different databases, underscoring that this is not a one-size-fits-all problem.

Following this benchmarking, the authors determined which genomes encoded the proteins of interest by choosing the most reliable HMM profiles for the protein type and removing false positives based on phylogenetic similarity to the negative controls. Notably, this approach provided new insights into hopanoid production in the *Bacillota* by distinguishing between SHC and the related enzyme sporulenol cyclase, which does not produce hopanoids. The authors similarly refined the understanding of hopanoid production in the *Planctomycetota* by distinguishing between hopanoid- and sterol-producing strains. Parsing these enzymatic differences is a key strength of this approach, one that was only possible due to the availability of experimental studies for validation. Notably, the benchmarking confidence for HpnR was limited by the availability of validation studies in the literature, and the authors omitted the hopanoid-modifying enzymes HpnG, HpnI, HpnJ, HpnK, and HpnO from the benchmarking for similar reasons.

The authors go on to analyze the evolutionary relationships within each group of homologs, determining that hopanoid production, including production of C_35_ hopanoids, likely originated in marine alphaproteobacteria. They also correlate hopanoid production with other biological processes, including osmotic stress response, single-carbon substrate utilization, and nitrogen cycling. By combining these observations with studies of hopanoid-deficient mutants both in the literature and in this study, a biological role for hopanoids in osmotic stress tolerance and nitrite utilization emerges.

Finally, Lawrence and colleagues sought to identify novel hopanoid-related genes by searching the regions upstream and downstream of the *shc* gene in their annotated genomes. Through this analysis, they found four uncharacterized gene families, two of which had been previously associated with hopanoid production. Three of these gene families encode proteins predicted to be involved in lipid binding and transport: HpnM and two new proteins that the authors name HpnS and HpnT. Through phylogenetic analysis and computational binding predictions, they hypothesize that in most proteobacteria, HpnM and HpnS transport hopanoids across the outer membrane in a system analogous to the Mla pathway, whereas in rhizobiales HpnT performs this function. These findings offer important insights into hopanoid transport, beyond the previously identified inner-membrane transporter HpnN.

All of these conclusions are strengthened by the rigorous pipeline Lawrence et al. report for hopanoid biosynthesis gene annotation. Beyond the study of these specialized lipids, this work provides a thoughtful and detailed model for integrating literature-derived experimental validation with the ease of precomputed HMM profiles.
